# Trait impulsivity is not related to post-commissural putamen volumes: A replication study in healthy men

**DOI:** 10.1371/journal.pone.0209584

**Published:** 2018-12-20

**Authors:** Fernando Caravaggio, Pontus Plavén-Sigray, Granville James Matheson, Eric Plitman, M. Mallar Chakravarty, Jacqueline Borg, Ariel Graff-Guerrero, Simon Cervenka

**Affiliations:** 1 Research Imaging Centre, Centre for Addiction and Mental Health, Toronto, Ontario, Canada; 2 Department of Psychiatry, University of Toronto, Toronto, Ontario, Canada; 3 Department of Clinical Neuroscience, Center for Psychiatry Research, Karolinska Institutet and Stockholm County Council, SE, Stockholm, Sweden; 4 Department of Biological & Biomedical Engineering, McGill University, Montreal, Quebec, Canada; 5 Cerebral Imaging Centre, Douglas Mental Health Institute, McGill University, Montreal, Quebec, Canada; 6 Department of Psychiatry, McGill University, Montreal, Quebec, Canada; University of California, San Francisco, UNITED STATES

## Abstract

High levels of trait impulsivity are considered a risk factor for substance abuse and drug addiction. We recently found that non-planning trait impulsivity was negatively correlated with post-commissural putamen volumes in men, but not women, using the Karolinska Scales of Personality (KSP). Here, we attempted to replicate this finding in an independent sample using an updated version of the KSP: the Swedish Universities Scales of Personality (SSP). Data from 88 healthy male participants (Mean Age: 28.16±3.34), who provided structural T1-weighted magnetic resonance images (MRIs) and self-reported SSP impulsivity scores, were analyzed. Striatal sub-region volumes were acquired using the Multiple Automatically Generated Templates (MAGeT-Brain) algorithm. Contrary to our previous findings trait impulsivity measured using SSP was not a significant predictor of post-commissural putamen volumes (β = .14, df = 84, *p* = .94). A replication Bayes Factors analysis strongly supported this null result. Consistent with our previous findings, secondary exploratory analyses found no relationship between ventral striatum volumes and SSP trait impulsivity (β = -.05, df = 84, *p* = .28). An exploratory analysis of the other striatal compartments showed that there were no significant associations with trait impulsivity. While we could not replicate our previous findings in the current sample, we believe this work will aide future studies aimed at establishing meaningful brain biomarkers for addiction vulnerability in healthy humans.

## Introduction

Heightened impulsivity is considered both a risk factor for, and a consequence of, chronic substance abuse and drug addiction [1–3]. Impulsivity is a multidimensional construct, encompassing impulsive choice, impulsive action, and self-reported impulsive personality traits [4]. While studies have not observed strong relationships between measures of impulsive choice and impulsive action, both are weakly correlated with measures of trait impulsivity [4–6]. Thus, elucidating the neural correlates of trait impulsivity may help inform models of drug addiction vulnerability [7, 8].

Several *in vivo* positron emission tomography (PET) studies have examined the neurochemical correlates of trait impulsivity in humans [9], with an emphasis on the striatal dopamine (DA) system [10]. Lower DA D_2/3_ receptor (D_2/3_R) availability, particularly in the ventral striatum (VS), has been associated with both drug-use and impulsivity in animals [11–15] and in people with drug addiction [16–18]. However, whether higher trait impulsivity is related to lower D_2/3_R availability in the VS of healthy humans remains unclear. While studies have differed in terms of the radiotracers and personality scales employed, there have been reports of no correlations [19–21], positive correlations [22, 23], as well as negative correlations [24, 25] between striatal D_2/3_R availability and impulsivity. These mixed findings do not lend strong support to the hypothesis that VS D_2/3_R availability may be a link between impulsivity and drug addiction [10].

We recently observed a negative correlation between D_2/3_R availability in the VS and trait impulsivity, measured using the Karolinska Scales of Personality (KSP), in healthy people [24]. Since lower D_2/3_R availability in the VS has been related to smaller VS volumes in rodents [26] and humans [27, 28], we explored whether trait impulsivity measured with the KSP was related to VS volumes in healthy humans [29]. While trait impulsivity was not significantly associated with VS volumes, in a *post-hoc* analysis we observed a significant sex-interaction in the post-commissural putamen. Specifically, greater impulsivity was associated with smaller post-commissural putamen volumes in males, but not in females [29].

Overall, studies examining the relationship between impulsivity and striatal volumes in healthy persons have provided mixed results [30–33]. These studies have employed various measures, sample sizes, and age ranges. Regarding impulsive choice (i.e. delay discounting), negative correlations with post-commissural putamen volumes (n = 34) [31], positive correlations with caudate volumes (n = 70) [32], and positive correlations with VS volumes in adolescents (n = 1830) [33] have been observed. Also, trait negative urgency (the tendency to act rashly under extreme negative emotions) has been related to smaller left ventral striatum volumes after controlling for other potentially confounding traits: neuroticism (negative emotionality), sensation seeking, and lack of planning and perseverance (n = 152) [30]. While our observed sex-interaction between post-commissural putamen volumes and trait impulsivity was unexpected [29], this work warranted replication for several reasons. First, if the effect is robust, it could potentially reconcile previously reported mixed-findings in the literature between trait impulsivity and striatal volume. Second, it could further inform biological factors underlying sex-differences in substance abuse vulnerability.

In the current investigation, we attempted to replicate our previous findings between trait impulsivity and post-commissural putamen volumes in a larger sample of healthy Swedish men, using the Swedish Universities Scale of Personality (SSP) [34], an updated version of the KSP. Specifically, our *a priori* hypothesis was that trait impulsivity would be negatively correlated with post-commissural putamen volumes in healthy men. To help us further interpret our *a priori* findings, secondary exploratory analyses were conducted examining potential laterality effects as well as potential relationships between trait impulsivity and VS volumes. Collectively, this work will help to further clarify how striatal morphology may relate to trait impulsivity in humans.

## Materials and methods

### Participants

Data from 88 healthy male participants was included in this replication study (Table 1). Specifically, structural T1-weighted images and self-report measures of trait impulsivity data were pooled from various PET studies [35–38]. All studies, and their design, were approved by the Regional Ethics Committee in Stockholm and the Karolinska University Hospital Radiation Safety Committee. All subjects gave written informed consent prior to participating according to the Helsinki declaration.

**Table 1:** Participant demographics (n = 88, male).

	Age	Trait Impulsivity– SSP	Total Brain Volume	Post-Commissural Putamen Volume
**Mean ±S.D.**	28.16 ±3.34	50.84 ±9.29	1584685.86 ±101878.52	3781.23 ±372.50
**Range:**	21.32–34.96	29.48–78.85	1369636–1872065	2957–4751

Structural data for 76 subjects was acquired on a 1.5 T GE Signa system (Milwaukee, WI) (hereafter termed Scanner 1) and for 12 subjects on a 1.5T Siemens Magnetom Avanto system (Erlangen, Germany) (hereafter termed Scanner 2). Exclusion criteria for all subjects included historical or present episode of psychiatric illness, alcohol or drug abuse, major somatic illness, or habitual use of nicotine as determined by a physical and psychiatric examination by a physician.

### Swedish Universities Scales of Personality (SSP)

All participants completed either the KSP196 questionnaire (N = 54) or the Swedish Universities Scales of Personality (SSP) (N = 34) [34]. KSP196 and SSP are updated versions of the original KSP [39] employed in our previous studies [24, 29]. The SSP shows improved psychometric properties, as well as updated normative data for healthy samples [34]. The SSP impulsivity scale was derived from the KSP196 impulsivity scale. This measure of trait impulsivity denotes the degree to which subjects’ report that they act on the spur of the moment (non-planning impulsivity). Responses are made on a four-point Likert scale (“does not apply” to “applies completely”), and includes statements such as, “I have a tendency to act on the spur of the moment without really thinking ahead.” Notably, the impulsivity subscale on the KSP and KSP196 includes 10-items (Cronbach's α = .68), while the SSP includes 7 items showing slightly higher internal reliability (Cronbach's α = .73) [34]. In the creation of the SSP impulsivity scale, three of the original items were removed: KSP-20, KSP-62 and KSP-113. The phrasing of two other items was also slightly changed (KSP-8/SSP-5 and KSP-68/SSP-44). In general, the reasons for the rephrasing was to enhance clarity and replace outdated words/phrasing. The impulsivity scales of the KSP and SSP are highly correlated with one another (r = 0.89, *p* < 1 x 10^−15^), based on data from [40].

### Magnetic resonance imaging (MRI)

The parameters for scanner 1 were as follows: T1-weighted imaging, TE = 5 ms, TR = 20 ms, 3D, 256 x 256, voxel-size 1 mm isotropic. The parameters for scanner 2 were as follows: T1-weighted imaging, TE = 3.53 ms, TR = 1.79 seconds, 3D, 256 x 256, voxel-size 1 mm isotropic.

### Subcortical volume analyses

**Fig 1:**
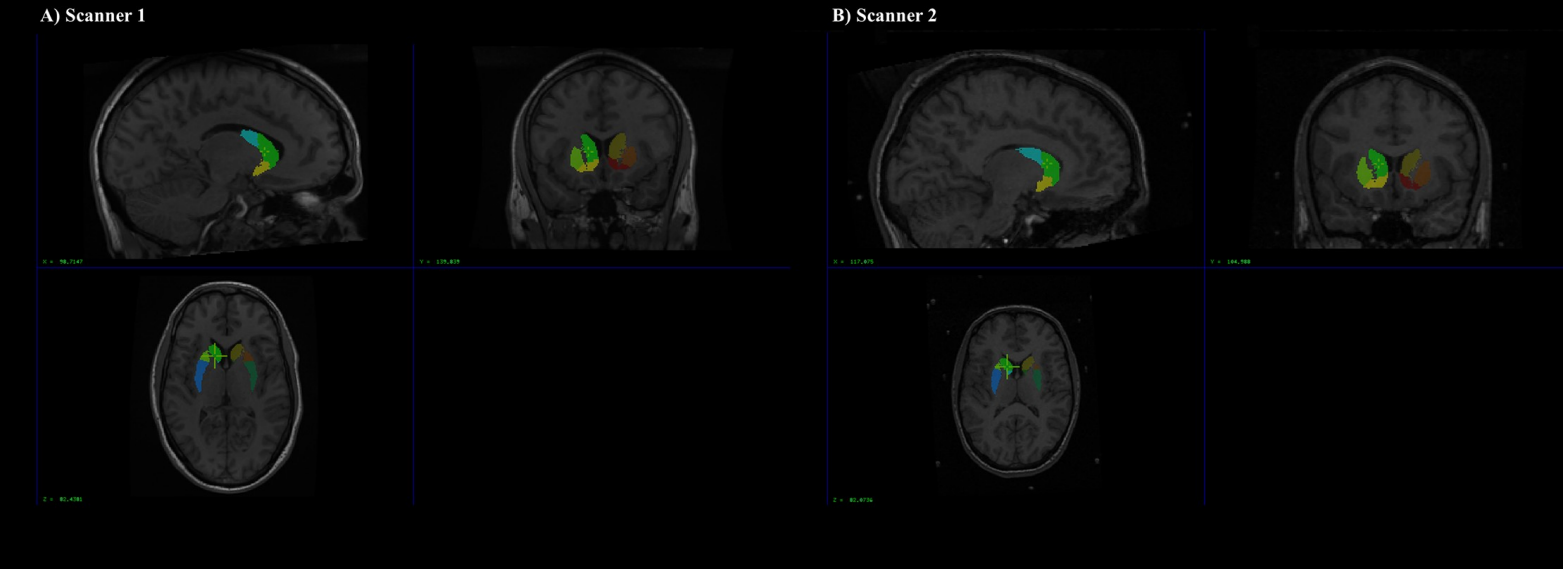
Regions of interest. Region of interest labels in native space produced by MAGeT-Brain in a randomly selected subject from Scanner 1 (A) and Scanner 2 (B).

The Multiple Automatically Generated Templates (MAGeT-Brain) algorithm [41, 42] was employed to provide fully-automated segmentation of striatal subdivisions (Fig 1) [43]. The delineation of the striatal subdivisions based on serial histological data, and the Collin27 Brain atlas (http://www.bic.mni.mcgill.ca/ServicesAtlases/Colin27), has been described in detail elsewhere (https://github.com/CobraLab/atlases) [43]. These include the pre-commissural caudate, post-commissural caudate, pre-commissural putamen, post-commissural putamen, and the VS. Briefly, the putamen (and caudate) can be divided based on the position of the anterior commissure as seen on the coronal plane. The portion of the putamen located anteriorly to the coronal location of the anterior commissure is demarcated as pre-commissural. The remaining portions of the putamen posterior to the anterior commissure, is considered post-commissural [44, 45]. Several studies have been conducted to validate the reliability of MAGeT-Brain against “gold-standard” manual segmentation–the correlation between methods in the striatum have been reported to be around r = .92 (*p* = .0001) with a DiceKappa of 0.861 [41, 42, 46–48]. Typically, in a multi-atlas segmentation approach, manually drawn labels from atlases are warped (or propagated) into native subject space by applying transformations estimated from non-linear image registration. Candidate labels from all atlas images are fused (via probabilistic segmentation techniques) to create a final segmentation. The goal of the MAGeT-Brain algorithm is to mitigate sources of error from regular multi-atlas segmentation approaches, including: 1) spurious non-linear registration or resampling errors (including partial volume effects in label resampling), and 2) irreconcilable differences in neuroanatomy between the atlas and target images. The MAGeT-Brain algorithm is a modified multi-atlas segmentation technique, which employs a limited number of high-quality manually segmented atlases as an input to reduce bias and enhance segmentation accuracy. MAGeT-Brain propagates atlas segmentations to a template library, formed from a subset of target images, via transformations estimated by nonlinear image registration. The resulting segmentations are then propagated to each target image and fused using a label fusion method.

Templates images for Scanner 1 (n = 21) and Scanner 2 (n = 11) were chosen as random sub-samples of the full-samples. These randomly selected cases were used as a template library through which the final segmentation was bootstrapped. Each subject in the template library was segmented through non-linear atlas-to-template registration followed by label propagation, yielding a unique definition of the subdivisions for each of the templates. The bootstrapping of the final segmentations through the template library produces candidate labels for each subject, and the labels are then fused using a majority vote to complete the segmentation process. Since this is a majority vote process, to avoid potential “ties” an odd number of template images were employed. Non-linear registration was performed using a version of the Automatic Normalization Tools (ANTS) registration technique [49] that is compatible with the minc toolkit (https://github.com/vfonov/mincANTS).

The effects of using multiple input atlases, varying the size of the template library constructed, has been rigorously examined for MAGeT-Brain previously [42]. Given the high computational demands of multi-atlas techniques, the MAGeT-Brain method is predicated on the finding that a useful template library can be generated from a small set of labelled atlas images [42]. While increasing the number of templates used improves the MAGeT-Brain segmentation, using even a smaller number of atlases (as low as n = 21 for samples ≥22) greatly improves the overlap between manually generated “gold standard” segmentations and automatically generated segmentations [46, 50]. Thus, while we have used smaller template libraries than the full sample, our segmentations are improved compared to other automated segmentation approaches [46].

Volumes (mm^3^) from ROIs were averaged across hemispheres. Compared to other automated techniques such as FreeSurfer and FSL, MAGeT-Brain demonstrates the highest correlation with gold-standard manual segmentation techniques, while FreeSurfer and FSL significantly overestimate subcortical volumes compared to MAGeT-Brain [46]. Quality control by visual inspection was carried out by authors FC and EP to ensure that, 1) there were no major artifacts in the original T1 images, and, 2) no anomalies in the labelling of the subcortical structures by examining for each subject the original subject image with the resulting labelled image.

### Total brain volume analysis

Total brain volume (TBV) was obtained using the Brain Extraction based on non-local Segmentation Technique (BEaST) method [51]. This method is based on non-local segmentation in a multi-resolution framework. Each voxel is labeled based on the similarity of its neighborhood of voxels to all the neighborhoods in a library of pre-defined priors, and a non-local means estimator is used to estimate the label at the voxel. Inputs are down-sampled to a lower resolution, segmentation is performed, and results are propagated up to higher resolutions [51]. BEaST is designed to include CSF (in the ventricles, cerebellar cistern, deep sulci, along surface of brain, and brainstem), the brainstem, and cerebellar white matter (WM) and gray matter (GM) in the brain mask, while excluding the skull, skin, fat, muscles, dura, eyes, bone, exterior blood vessels, and exterior nerves.

### Statistical analysis

We conducted *a priori* as well as complementary secondary exploratory analyses. For our *a priori* analysis, the hypotheses tested were:

1. H1: A negative relationship between post-commissural putamen volume and SSP impulsivity.
2. H0: No relationship between post-commissural putamen volume and SSP impulsivity.

We employed a linear-mixed-effects (LME) model, taking the hierarchical structure (subjects belonging to two different scanner groups) into account. The post-commissural putamen volumes were specified as the dependent variable, SSP impulsivity as the independent variable, and TBV and age as co-variates. Scanner condition (‘Scanner 1’ or ‘Scanner 2’) was specified as a random effect, allowing the intercepts to vary. All continuous variables of interest were standardized (z-scored) before being entered into the statistical model. We examined that the assumptions of linear regression were not violated our analyses. Alpha for this *a priori* test was set to 0.05 (one-sided expecting a negative relationship).

A *p*-value in-and-of itself is often a poor metric for assessing the success of a replication attempt, since the difference between a “significant” and a “non-significant” *p*-value is not necessarily meaningful [52]. For this reason, a statistical procedure known as the replication Bayes Factor (BF) [53] was also employed. A BF quantifies the relative strength of evidence in favor of two hypotheses by computing the predicative adequacy of H1 over H0 relative to one another. For the correlation replication BF specifically, H1 is defined as the posterior distribution of the correlation coefficient from the original study, assuming that a uniform prior was employed, and H0 is defined as a point null hypothesis of no effect [54]. Correlation coefficients were obtained by converting the test-statistics of the original finding (KSP Impulsivity, *r* = -.62) and the test-statistics from the abovementioned LME model to correlation coefficients [55]. The BF was calculated using the Savage-Dickey ratio [56]. A BF above 3 for H1 (BF10 > 3 or BF01 < 1/3) is commonly interpreted as providing moderate evidence for a successful replication, and a BF above 3 for H0 (BF01 > 3 or BF10 < 1/3) as moderate evidence for a failed replication. A BF above 10 indicates strong evidence in favor of one hypothesis (H1 or H0), over the other.

We also conducted secondary *post hoc* exploratory analyses to help inform our *a priori* findings. Specifically, we explored whether there were relationships between the left versus the right post-commissural putamen volumes and SSP impulsivity. Finally, in accordance with the previously reviewed literature, we examined whether VS volumes were related to SSP impulsivity (average ROI, as well as left and right ROIs separately). All statistical modelling was carried out using R (v.3.3.2).

## Results

### A priori analysis

**Fig 2:**
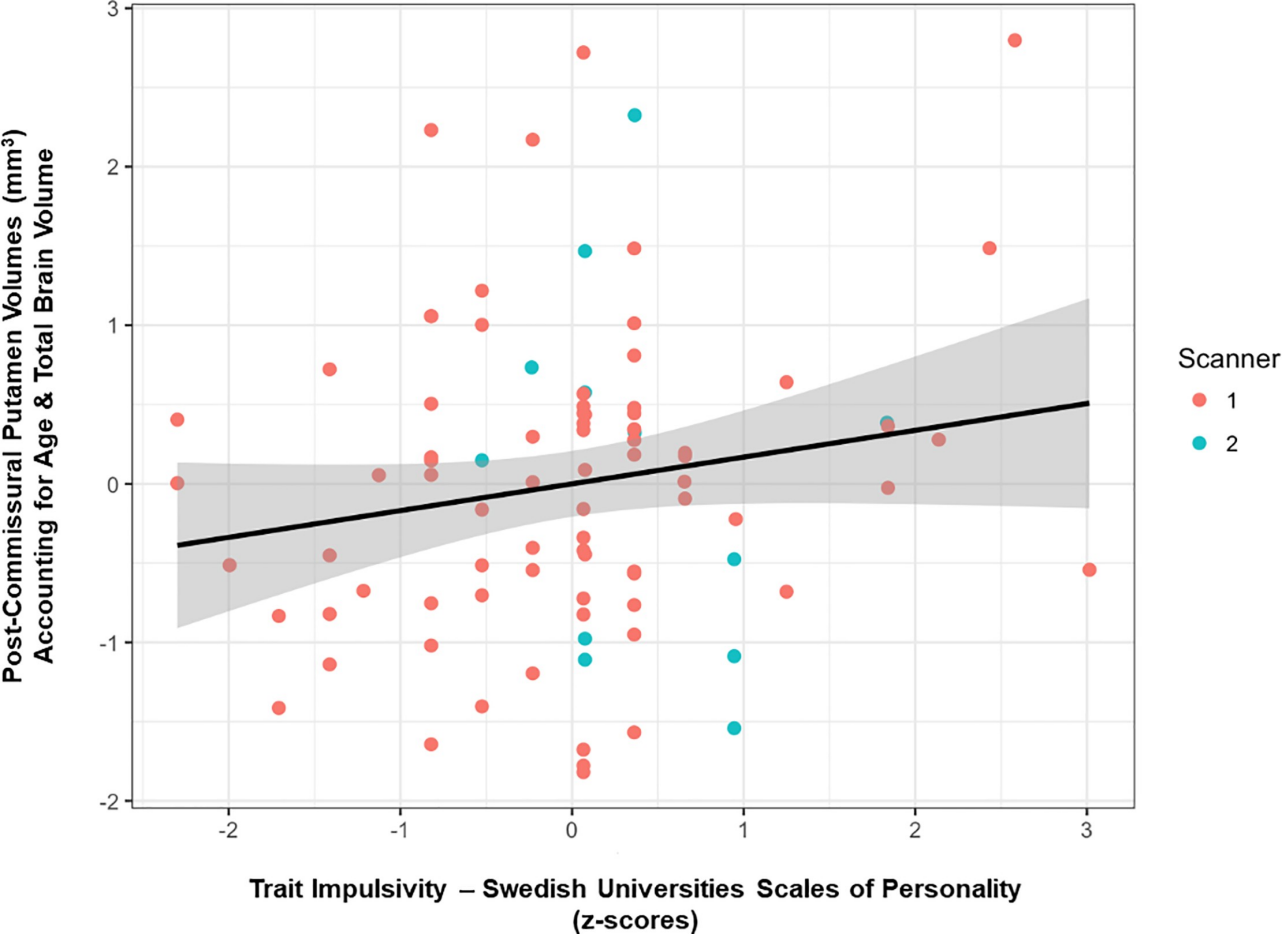
Trait impulsivity and post-commissural putamen volumes. The relationship between trait impulsivity measured by the Swedish Universities Scales of Personality (SSP) and post-commissural putamen volumes in 88 healthy males. Post-commissural putamen volumes have been corrected for age and total brain volume, and SSP impulsivity scores have been standardized (z-scored). The shaded area represents the 95% confidence interval.

**Fig 3:**
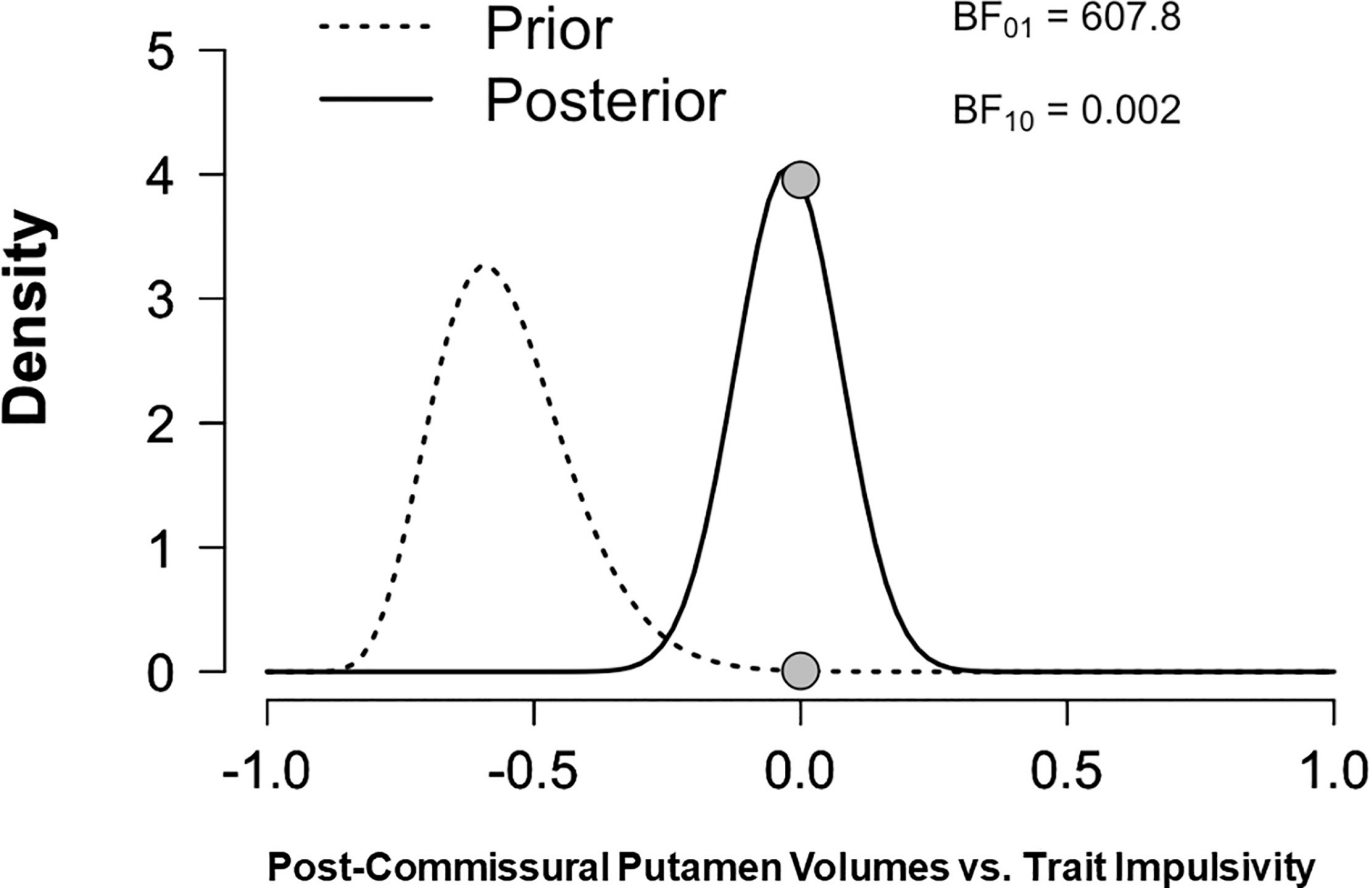
Replication bayes factor analysis. The results of the replication Bayes Factor (BF) analysis. The dashed line (the “prior”) denotes the original relationship (previously published) between post-commissural putamen volumes and trait impulsivity, and the updated estimate (“the posterior”) from data from the current study. BF was calculated using the ratio between the heights of the prior and posterior at zero (denoted by the grey circles). The label “Density” on the y-axis refers to the probability density.

Contrary to our previous findings [29], trait impulsivity measured by the SSP was not a significant predictor of post-commissural putamen volumes (β = .14, df = 84, *p* = .94) (Table 2 & Fig 2). Table 3 outlines the beta-weights compared between the previous publication [29], the current findings, as well as the replication BF. The replication BF in favor of H0 (a failed replication) was 607.8 and inversely, the replication BF in favor of H1 (a successful replication) was 0.0016. Hence, replication BFs showed that the data was over 607 times more likely to have occurred under H0, compared to under that of the posterior distribution of the original study, i.e. a strong negative relationship between trait impulsivity and post-commissural putamen volume (Fig 3).

**Table 2:** Simple liner-mixed effects (LME) model predicting post-commissural putamen volumes.

	Variables	Estimate (β)	*t*-value	*p*-value
*Independent*:				
	*Trait Impulsivity–SSP*	.14	1.60	.94
*Covariates*:				
	*Age*	-.33	-3.91	.0002
	*Total Brain Volume*	.49	5.65	.0000002

All variables of interest were standardized (z-scored) before entering the statistical model.

**Table 3:** Data used for calculating replication Bayes function.

Study	Beta (β)	Degrees of Freedom	*p*-value^†^	BF10	BF01
*Original*	-.62	27	.0003	-	-
*Current*	.14	84	.94	.0016	607.8

^†^The original analysis was two-tailed. The current analysis was a one-tailed test in the direction of the original study.

### Post hoc exploratory analyses

First, excluding subjects imaged with Scanner 2 (n = 12) did not significantly change our *a priori* null findings (β = .16, df = 72, *p* = .96). Second, trait impulsivity measured by the SSP was not a significant predictor of either left (β = .12, df = 84, *p* = .92) or right (β = .15, df = 84, *p* = .96) post-commissural putamen volumes (Table 4). Further exploratory analyses demonstrated that trait impulsivity measured by the SSP was also not a significant predictor of VS volumes (Table 5). A further exploration of all the other striatal subdivisions is presented in Table 6 (two-tailed tests). Notably, pre-commissural caudate volumes were negatively associated with trait impulsivity measured with the SSP (β = -.20, *t* = -2.15, *p* = .03). However, this relationship did not survive Bonferroni correction for multiple comparisons (corrected *p*-threshold = .006).

**Table 4:** Results of secondary *post hoc* analyses exploring potential effects of laterality.

Region of Interest	Variables		Estimate (β)	*t*-value	*p*-value^†^
*Left Post-Commissural Putamen*					
	*Independent*:				
		*Trait Impulsivity—SSP*	.12	1.43	.92
	*Covariates*:				
		*Age*	-.33	-3.91	*p* < .0001
		*Total Brain Volume*	.49	5.68	*p* < .0001
*Right Post-Commissural Putamen*					
	*Independent*:				
		*Trait Impulsivity—SSP*	.15	1.71	.96
	*Covariates*:				
		*Age*	-.33	-3.73	*p* < .0001
		*Total Brain Volume*	.47	5.35	*p* < .0001

^†^Results are reported as one-tailed tests.

**Table 5:** Results of secondary *post hoc* analyses exploring potential relationships between trait impulsivity and ventral striatum volumes.

Region of Interest	Variables		Estimate (β)	*t*-value	*p*-value^†^
*Ventral Striatum*					
	*Independent*:				
		*Trait Impulsivity—SSP*	-.05	-.58	.28
	*Covariates*:				
		*Age*	-.21	-2.34	.02
		*Total Brain Volume*	.48	5.21	.000001
*Left Ventral Striatum*					
	*Independent*:				
		*Trait Impulsivity—SSP*	-.04	-.41	.34
	*Covariates*:				
		*Age*	-.25	-2.77	.01
		*Total Brain Volume*	.48	5.24	.001
*Right Ventral Striatum*					
	*Independent*:				
		*Trait Impulsivity—SSP*	-.05	-.55	.29
	*Covariates*:				
		*Age*	-.19	-1.91	.06
		*Total Brain Volume*	.45	4.71	.001

^†^Results are reported as one-tailed tests.

**Table 6:** Results of secondary post hoc analyses exploring potential relationships between trait impulsivity and all the remaining striatal subdivisions.

Region of Interest	Variables		Estimate (β)	*t*-value	*p*-value^†^
*Pre-Commissural Putamen*					
	*Independent*:				
		*Trait Impulsivity—SSP*	-0.05	-0.58	.56
	*Covariates*:				
		*Age*	-0.21	-2.34	.02
		*Total Brain Volume*	0.48	5.21	>.0001
*Pre-Commissural Caudate*					
	*Independent*:				
		*Trait Impulsivity—SSP*	-0.20	-2.15	.03
	*Covariates*:				
		*Age*	-0.34	-3.66	.0004
		*Total Brain Volume*	0.33	3.60	.0005
*Post-Commissural Caudate*					
	*Independent*:				
		*Trait Impulsivity—SSP*	-0.10	-1.18	0.24
	*Covariates*:				
		*Age*	-0.49	-5.82	>.0001
		*Total Brain Volume*	0.34	3.95	.0002

^†^Results are reported as two-tailed tests.

## Discussion

Determining the neural correlates of impulsivity in healthy persons may help inform biological markers of drug addiction vulnerability. Research on how trait impulsivity may be related to striatal neurochemistry and morphology have yielded mixed results [30–33]. In the current investigation, we attempted to replicate our previous observation that higher trait impulsivity was related to smaller post-commissural putamen volumes in healthy men [29]. Contrary to our previous findings, we observed no significant relationship between trait impulsivity and post-commissural putamen volumes in a larger, independent sample of healthy males. Rather, we found strong evidence in favor of a failed replication: the data were over 607 times more likely to have occurred under the null hypothesis of no effect than they were under the outcome of the original study. Furthermore, in line with our initial study, our exploratory analyses did not show a significant relationship between trait impulsivity and VS volumes in healthy males.

It is difficult to definitively interpret the results of a failed replication, and there were several differences between the studies which could be speculated to have led to the differing results. First, the original investigation employed a statistically significantly older sample than the current investigation (Mean Age: 32.13±9.13 versus 28.16±3.34; *t* = 3.49, df = 117, *p* = .0007). Thus, it is possible that trait impulsivity may be negatively correlated with post-commissural putamen volumes in older (≥34 years of age) but not younger subjects. However, we are currently unaware of any evidence *a priori* to support this potential interpretation. Thus, we do not suspect that this age difference significantly accounts for the observed discrepancy between studies. Second, the current investigation employed an updated scale measuring trait impulsivity compared to the original investigation. However, the KSP and SSP Impulsivity scales are in theory meant to measure the same construct [34]. In a separate sample of 304 individuals who filled out both the KSP and SSP [40], the correlation between the two measures was found to be very high. It is therefore highly unlikely that the removal of three out of ten items, which was done to improve the reliability, could explain the different results. Moreover, it cannot be fully excluded that differences in genetic, cultural, and socioeconomic measures between the Canadian and Swedish samples could have lead to discrepancies between studies, and these factors should be taken into consideration in future investigations. Finally, different sequence parameters for the T1-image acquisition were employed between the previous study and the current investigation. Since these parameters (TR, TE, flip angle) influence image contrast, it is possible that there were slight differences in tissue classification and segmentation between studies [57], which in turn may have contributed to our differing results. While this potential effect can not be directly examined by our current data, it is important to note that the MAGeT-brain method shows both a high degree of test-retest reliability and congruence with “gold-standard” manual segmentation techniques [46].

There are several strengths and weaknesses associated with the current investigation. First, this study employed a substantially larger sample of healthy males compared to our initial study (n = 88 versus n = 31). Second, we employed an improved measure of trait impulsivity compared to the original investigation. However, like our original investigation, this study was retrospective. Therefore, the influence of other important demographic and psychological measures relevant to trait impulsivity and striatal morphology could not be investigated. For example, several lines of evidence suggest that motivational deficits may be related to both striatal dopaminergic functioning and striatal morphology in healthy persons [58–61] and persons with neuropsychiatric diseases [61–63]. Therefore, future studies should examine the potential interactions between trait impulsivity and motivational functioning on striatal morphology; for instance, using behavioural measures specifically designed to dissociate these related traits and behaviours [64, 65]. Moreover, there may be other participant differences–such as lifestyle factors and other personality traits (e.g. social desirability)–which may have lead to the differing results between the Toronto and Karolinska samples. Unfortunately, such influences cannot be readily determined from the data collected from both samples. Finally, our investigation only examined male participants. Future studies should examine the relationship between trait impulsivity and striatal morphology in larger samples of both healthy men and women.

We attempted to replicate a previously observed negative association between trait impulsivity and post-commissural putamen volumes in healthy males, finding strong evidence in favor of a failed replication. Especially in neuroscience, there is a growing need for more replication attempts in larger independent samples before strong research claims can be made [66, 67]. Neuroscience as a field particularly suffers from a lack of replicability for several reasons. First, surveys suggest neuroscience studies in general are underpowered [67]. Low statistical power overestimates the true effect sizes of observed findings, resulting in these findings being less likely to be reproduced. While unexpected findings from smaller samples–like in our original study (n = 31)–can point to true, strong effects [68], replication in multiple, larger samples is the only means of validating these findings [69]. Large variations in neuroimaging methods further decrease replicability [70], with the majority of surveyed studies reporting associations between structural brain morphology and behaviour failing to replicate [71]. Collectively, this crisis in replicability severely impairs the progress of neuroscience as a field [72]. We believe our replication attempt represents an important step towards such goals, and we hope that future studies building upon this line of research will help establish new meaningful brain biomarkers for addiction vulnerability in healthy humans.
